# Progress in Surgery

**Published:** 1895-05-18

**Authors:** 


### PROGRESS IN SURGERY.

### SURGERY OF THE ŒSOPHAGUS AND STOMACH.

**Stricture of the Œsophagus** has been successfully divided with a string (Abbe's method) after gastrotomy by Murray[1] in a case of impassable stricture caused by swallowing a quantity of caustic potash a year before. A similar case is also reported by Mayo.[2] Mansell Moullin[3] has also successfully treated a case by gastrotomy and retrograde dilatation after the manner devised by Loreta. According to Kendal Franks this operation has been performed twenty-one times in all for fibrous stricture of the œsophagus, and not one of them was fatal. Kendal Franks[4] has treated one of those peculiar cases known as simple stenosis of the œsophagus by œsophagoctomy. On the belief that the dysphagia was due to a papillomatous growth, he opened the œsophagus on the left side but found the stenosis was formed of healthy mucous membrane, without any cicatricial tissue. The appearances were as if a string had been passed beneath the mucous membrane, completely round the tube and had been tied tight; but, on the other hand, there was no wrinkling of the mucous membrane, which was perfectly smooth. He divided the mucous membrane transversely above and below, round the whole circumference of the tube, and excised the middle portion completely, so that between the mucous membrane above and the mucous membrane below there was a ring of raw muscular tissue about an inch and a half wide. The mucous membrane above was sutured to the mucous membrane below, a tube was passed through the nose, down the œsophagus and into the stomach and the longitudinal incision in the œsophagus brought together with stitches, and the external wound closed. Œsophagoctomy has hitherto been performed six times, and always for malignant disease. The number of cases recorded of simple stenosis is only nine, in addition to that here given. Of these ten cases, five occurred at the upper extremity of the œsophagus, immediately below the cricoid cartilage (as in the author's case), four were found at the cardiac end, and in one (Baillie's) the situation is not given. Most writers attribute these strictures to a congenital abnormality, but the history rarely bears out this assumption, and Franks thinks it is more likely due to a very localised and prolonged spasmodic condition of the muscularis mucosæ, or of the circular muscular fibres of the outer coat. How this is brought about is difficult to say, but Harrison Cripps[5] has described a somewhat analogous condition in the rectum.

**Œsophagitis Gangrenosa.**—Pietkiewiez[6] describes the case of a labourer, aged 25, who swallowed some caustic soda by mistake. A purulent inflammation of the submucous cellular tissue of the œsophagus ensued, undermining the whole mucous membrane, causing necrosis and detachment, so that on the eleventh day he coughed out a cast of the gullet in the form of a cylinder 22 centimetres long, from 2½ to 3½ centimetres broad, and from 1 to 2 millimetres in thickness. About six months after the accident the man died of exhaustion.

**Intrathoracic Œsophagotomy.**—Potarca[7] describes this operation in the posterior mediastinum which he has performed on the dead subject. The body being turned over on the belly, a vertical incision, between five and six inches in length, the middle of which corresponds to the fourth dorsal spine is made midway between the internal boder of the scapula and the spines of the dorsal vertebræ. The following structures are divided: the aponeurosis of the trapezius, and some of its muscular fibres at the lowest part of the wound, the aponeurosis and lower fibres of the rhomboideus major, the aponeurosis between the two serrati postici muscles; then after separation of the sacro-lumbales from the longissimus dorsi, the transversalis colli. The third, fourth, and fifth ribs having been thus exposed, a piece of bone about an inch in length is removed from each, the internal section of each rib being close to the transverse process of the corresponding vertebræ. The pleura is now carefully stripped from the inner fragments of the ribs, and the front of the spine, and the vena azygos major exposed, in front of which will be seen the œsophagus at a depth of four inches from the wound in the skin. A foreign body, previously introduced for this purpose, has been extracted from the œsophagus by this deep wound, and the incision in the œsophagus closed by six sutures.

**Diverticulum of the Œsophagus.**—Konig[8] describes two cases in patients aged 50 and 62 respectively, in which such a diverticulum was successfully removed. The sac in both cases sprang from near the lower limit of the pharynx. In both dysphagia was present. Food could be got into the stomach when the sac was full, but this was impossible after a time owing to the size of the sac. No sound could be passed into the stomach. The enucleation of the sac was fairly easily effected by the fingers. The neck of the sac in each case was about as thick as the thumb. Two sets of sutures, one through the mucous membrane, the other through the outer wall, were used, while the neck of the sac was gradually divided from below upwards.

**Gastro-enterostomy.**—Hahn[9] has lately performed fifteen operations of this kind, and all have survived, In cases of extensive resection of the stomach, he considers it undesirable to attempt to unite the stomach and duodenum, but a gastro-jejunostomy should be done after sewing up the stomach and duodenum. The opening in the stomach should be near the pylorus, and that in the intestine near the attachment of the mesentery. Rosenheim[10] has examined 10 cases of gastro-jejunostomy, and has nearly always found, whether the primary disease was malignant or not, delay in the passing on of the stomach contents. Complete restitution of the stomach mechanism has not hitherto been observed.

**Gastrotomy for Removal of Foreign Bodies.**—Mayo Robson[11] successfully removed from the stomach of a girl aged ten 42 cast-iron nails, 1⅝ in. long; 93 brass and tin tacks, from ½ in. to 1 in. long; 12 large nails, some brass-headed; three collar studs, one safety pin, and one sewing needle. The child confessed to swallowing nails since 1893. A somewhat similar case is recorded by Gemmel[12] in a lunatic, aged forty-three, from whose stomach he removed 192 large nails and other articles, weighing in all 1 lb. 9½ oz.

**Pyloroplasty.**—Miller[13] has operated successfully on a case of spasmodic stricture of the pylorus in a woman aged forty-eight, after the method of Heineke-Mikulicz; and a similar result attended the same operation performed by Markoe[14] on a man aged forty-nine, affected with cicatricial stenosis of the pylorus.

**Gastro-plication or Stomach-reefing.**—Brandt (Klausenburg)[15] has performed this operation for dilatation of the stomach, in which no definite cause was found for the gastrectasia after opening the abdomen. The patient was a woman twenty-six years of age. The pylorus was especially examined, and nothing found. The stomach was enormously enlarged. He proceeded to fold in the anterior wall and suture it by two rows of transverse sutures. The same was done on the posterior wall through holes torn through the great omentum. More than two hundred sutures were applied. The patient made an excellent recovery, without any disturbance of digestion, and was able to leave her bed on the tenth day.

**Gastropexy.**—Duret[16] relates the case of a woman, fifty-one years of age, who suffered from enteroptosis, with dilatation and descent of the stomach into the infra-umbilical region. All medical measures having failed, gastropexy was performed, the stomach being attached by its anterior wall to the abdominal parietes. After this the patient markedly increased in weight. This observation shows that in cases of prolapse of the stomach gastropexy is an efficient method of treatment, both with reference to the fixation of the stomach and cessation of the majority of the morbid symptoms.

**Gastrectomy.**—Langenbuch[17] relates two cases of almost complete resection of the stomach for carcinoma. (1) A woman, aged 58, much wasted, presented a kidney-shaped tumour in the hypochondrium. The malignant disease was found tc occupy chiefly the posterior surface. The stomach was drawn as much as possible out of the wound, and the operation conducted almost extraperitoneally. About seven-eighths of the stomach and some nodules in the omentum, were removed. The two ends of the stomach were then sewn together after the necessary narrowing of the cardiac end. The stomach then was the size of a hen's egg. The patient was seen in apparently perfect health 193 days after the operation. (2) In the second case of a woman, aged 56, the stomach was so soft that it tore during the removal. The rent was sewn up, but the patient died on the sixth day, chiefly from deficient nutrition. The author emphasises the following points: (1) The smallest amount (a few drops in the first case) of anæsthetic possible should be used; (2) all antiseptics should be avoided in the abdominal cavity; (3) the extraperitoneal method of resection should be adopted, the posterior part of the ring of sutures is surrounded by gauze, and the situation of the remaining sutures should be permanently extraperitoneal, the anterior wall of the stomach is covered in by a flap of skin; (4) the patient should be fed early by the mouth.

**Gastric Ulcer.**—Küster[18] reports a second case of gastric ulcer successfully treated by incision of the stomach, thermic cauterisation, and gastro-enterostomy in an emaciated man, aged 42, who for six years had suffered from frequent attacks of pain in the stomach and of vomiting, the vomit having contained blood for some months before operation. After opening the much dilated stomach a large deep ulcer with overhanging margin was found in the pyloric portion on the posterior wall. As the opening into the duodenum could not be found, an anastomosis was made with the duodenum. Küster concludes that: (1) Hæmorrhage from a gastric ulcer may be stopped by a single energetic application of the cautery; (2) gastro-enterostomy is to be preferred to pyloric resection in cases of gastric ulcer situated near the pylorus, as the latter does not preclude later distortion, or even occlusion; (3) an extensive union between the stomach and intestine is an advantage, as the circular line of suture always contracts. Cases of perforative gastric ulcer treated by operation (excision, stitching, &c.) are reported by Nicholson[19], Bennett[20], Eve[21], Morse[22], and others, with a good proportion of success. One of Bennett's cases was associated with a sub-phrenic pneumothorax. Ewald[23] thinks that in the presence of a simple gastric ulcer, surgical intervention is not advisable, except at very late date. It is known that in 85 per cent. of all cases of gastric ulcer recovery takes place without operation. Intervention should therefore be delayed, unless the patient suffers unbearable pain. Direct resection of the ulcerated part of the stomach results in recovery in 50 per cent. of such cases. Pariser[24] has collected statistics, mainly from English sources, which tend to show that, out of forty-three cases of round ulcer with perforation, ten recovered. He had a lady under his care some time since who suffered from acute peritonitis as the result of perforation of a gastric ulcer. As the stomach was empty when the perforation occurred he did not operate, but fed the patient exclusively through the rectum. She is now in perfect health.

[1] Annals of Surgery, Dec., 1894. [2] Therapeutic Gazette, July 16th, 1894, and North Western Lancet, Ap. 15th, 1894. [3] Lancet, Dec. 15th, 1894. [4] Brit. Med. Journ., Nov. 3rd, 1894. [5] Diseases of the Rectum and Anus, 2nd Edition. p. 224. [6] Brit. Med. Journ., Sept. 1st, 1894. [7] Brit. Med. Journ., Nov. 10th, 1894. [8] Berl. Klin. Woch., Oct. 15th, 1894, and Brit. Med. Journ., Nov. 10th, 1894. [9] Brit. Med. Journ., Nov. 17th, 1894, and Deut. Med. Woch., Oct. 25th, 1894. [10] Brit. Med. Journ., Jan. 5th, 1895, and Berl. Klin. Woch., Dec. 10th, 1894. [11] Lancet, Nov. 3rd, 1894. [12] Lancet, Aug. 25th, 1894, p. 432. [13] Lancet, Dec. 1st, 1894, p. 1,270. [14] Annals of Surgery, Dec., 1894, p. 740. [15] Annals of Surgery, Oct. 1894, and Central für Chirurg; No. 16th, 1894. [16] Med. Week., Oct. 5th, 1894, p. 488. [17] Brit. Med. Journ., Jan. 19th, 1895. and Deut. Med. Woch., Dec. 27th, 1894. [18] Brit. Med. Journ., Jan. 12th, 1895, Med. Chron., Sept, 1894, and Cent. f. Chir., No. 51, 1894. [19] Brit. Med. Journ., Nov. 3rd, 1894, and Dec. 22nd, 1894. [20] Lancet, Nov. 17th, 1894. [21] Lancet, Nov. 10th, 1894. [22] Therapeutic Gazette, Sept. 15th, 1894. [23] Med. Week., Nov. 3rd, 1894. [24] Med. Week., Dec. 21st, 1894.

